# Avoiding Arterial Hypotension in Preterm Neonates (AHIP)—A Single Center Randomised Controlled Study Investigating Simultaneous Near Infrared Spectroscopy Measurements of Cerebral and Peripheral Regional Tissue Oxygenation and Dedicated Interventions

**DOI:** 10.3389/fped.2018.00015

**Published:** 2018-02-01

**Authors:** Gerhard Pichler, Nina Höller, Nariae Baik-Schneditz, Bernhard Schwaberger, Lukas Mileder, Jasmin Stadler, Alexander Avian, Jasmin Pansy, Berndt Urlesberger

**Affiliations:** ^1^Research Unit for Neonatal Micro- and Macrocirculation, Department of Pediatrics and Adolescent Medicine, Medical University of Graz, Graz, Austria; ^2^Division of Neonatology, Department of Pediatrics and Adolescent Medicine, Medical University of Graz, Graz, Austria; ^3^Institute for Medical Informatics, Statistics and Documentation, Medical University of Graz, Graz, Austria

**Keywords:** near-infrared spectroscopy, cerebral oxygenation, peripheral-muscle oxygenation, centralization of circulation, early intervention

## Abstract

**Introduction:**

Up to 50% of preterm infants admitted to intensive care units require cardiocirculatory support. The aim of the present study was to assess whether simultaneous monitoring of cerebral tissue oxygenation index (cTOI) and peripheral tissue oxygenation index (pTOI) using near-infrared spectroscopy (NIRS) in combination with dedicated intervention guidelines may help avoiding arterial hypotension and catecholamine administration in preterm neonates.

**Study design:**

Preterm neonates <37 weeks of gestation were included in a single center randomized controlled study. Blood pressure was measured non-invasively or invasively. In the NIRS group, simultaneous cTOI and pTOI monitoring was used starting within 6 h after birth for 24 h to calculate changes in cTOI/pTOI ratio over time. Depending on these changes, interventions including echocardiography, administration of volume or patent ductus arteriosus treatment were performed. In the control group, only routine monitoring and treatment were performed and NIRS signals were not visible. The primary outcome was burden of hypotension within 48 h after initiation of NIRS monitoring.

**Results:**

49 preterm neonates were included in each group: NIRS group 33.1 (32.0–34.0) (median: 25–75 centile) weeks of gestation and control group 33.4 (32.3–34.3) weeks of gestation. In the NIRS group, echocardiography was performed in 17 preterm neonates due to NIRS measurements, whereby six neonates received further treatment. Percentage of neonates with any hypotensive episode during the 48-h observational period was 32.6% in the NIRS group and 44.9% in the control group (*p* = 0.214). Burden of hypotension (i.e., %mmHg of mean arterial pressure < gestational age) was 0.0 (0.0–2.1) mmHg h in the NIRS group and 0.4 (0.0–3.3) mmHg h in the control group (*p* = 0.313), with observed burden of hypotension being low in both groups. No severe adverse reactions were observed.

**Conclusion:**

In preterm neonates using simultaneous peripheral and cerebral NIRS measurements for early detection of centralization followed by predefined interventions led to a non-significant reduction in burden of arterial hypotension.

**Clinical Trial Registration:**

www.ClinicalTrials.gov, identifier: NCT01910467.

## Introduction

Arterial hypotension, irrespective of definition, occurs in up to 20% of preterm infants, most commonly during the first 48 h after birth (1). The management of hypotension in preterm infants includes volume administration, inotropes and corticosteroids in refractory cases. Very preterm infants in intensive care units receive fluid boluses in up to 50%, and/or pressor/inotrope medications for cardiovascular support, whereby the most frequent indication for intervention is a low systemic blood pressure in the first few days of life (1–4). Associations between systemic hypotension and fluctuations of blood pressure with cerebral injury such as intraventricular hemorrhage (IVH) (5, 6) and periventricular leukomalacia (PVL) (7, 8) have been described.

In early stages of shock, neonates often compensate for cardiovascular dysfunction and maintain normal blood pressure. The presence of hypotension is often a late finding (9, 10). Early cardiovascular and circulatory signs of inflammation especially in case of sepsis are difficult to interpret in neonates. In sepsis, microvascular dysfunction occurs secondary to perfusion heterogeneity, arteriovenous shunting and impaired autoregulation (11, 12). A correlation between microcirculatory abnormalities and organ dysfunction has already been shown (13). Conventional parameters of oxygenation and hemodynamic status may fail to detect microcirculatory dysfunction (14).

Near-infrared spectroscopy (NIRS) enables non-invasive measurement of oxygenation in regions of interest, e.g., cerebral, renal, and in peripheral muscle tissue (15). Most clinical work on cerebral oxygenation especially in term and preterm neonates has been undertaken using “continuous wave spatially resolved technique” NIRS (15–17).

Several studies have already investigated cerebral NIRS and cardiocirculation. Suresh et al. (18) found that cerebral fractional oxygen extraction changed at mean arterial blood pressure (MABP) levels below 23 mmHg, concluding that cerebral perfusion is probably maintained at MABP levels above 23 mmHg. In piglets, cerebral perfusion pressure increased immediately on norepinephrine, whereas cerebral oxygenation as reflected by tissue oxygenation index (TOI) did not improve, except by retransfusion (19).

In a recent study, we described associations between peripheral muscle NIRS parameters, MABP and heart rate (HR) in term and preterm neonates (20). Furthermore, in neonates with inflammatory processes and C-reactive protein (CRP) elevation NIRS measurements showed impaired peripheral oxygenation and perfusion when routine hemodynamic variables were still normal (9).

The primary aim of this study was to examine, whether it is possible to reduce arterial hypotensive episodes (MABP < gestational age, presented in %mmHg/h within the first 48 h after initiation of NIRS monitoring) and use of inotropes by applying simultaneous cerebral and peripheral muscle NIRS trend monitoring in combination with dedicated early clinical interventions. The secondary aim was to explore potential impact of these interventions on arterial hypotension, the use of inotropes as well as on cerebral injury and mortality.

We hypothesized that by using predefined clinical interventions in case of increase in cerebral/peripheral tissue oxygenation ratio (cTOI/pTOI), hypotensive episodes, and the use of inotropes could be reduced, which may result in less cerebral injury and mortality.

## Methods

This single center randomized controlled study (AHIP) was carried out at the Division of Neonatology, Medical University of Graz, Austria (EK-Nr: 25–237 ex 12/13; http://ClinicalTrials.gov Identifier: NCT01910467). Preterm neonates (<37 + 0 weeks of gestation) born after premature rupture of membranes, with amnion infection of the mother or increased maternal markers of systemic inflammation (CRP/leukocytes) were included. Written parental consent was obtained for all infants. Further inclusion criteria were decision to conduct full life support, age below 6 h after birth and no use of catecholamines before initiation of NIRS measurements. Exclusion criteria were any congenital malformations.

## Sample Size

For this randomized phase I/II study, the sample size was calculated at 49 neonates in each group resulting in a total number of 98 patients. Calculations were based on observations during the first 24 h after birth in 54 neonates. 15 of these neonates (27.8%) had episodes of hypotension (unpublished data). In the present study, participants were randomized into either the NIRS group or the control group. The ratio of allocation was 1:1. In case of multiple births, only the first infant was randomized.

## Interventions

After study inclusion, NIRS measurements with NIRO 200NX (Hamamatsu Photonics, Hamamatsu City, Japan) were performed in combination with routine monitoring (pulse oximetry, electrocardiography). Blood pressure was measured invasively, when an intra-arterial line was placed routinely and non-invasively with a pneumatic cuff placed around the left upper arm measuring at least every 30 min.

Invasive and nonivasive blood pressure measurements were performed with the IntelliVue MP50 monitor (Philips, Netherland). Data were stored in a polygraphic system (alpha-trace digitalMM, B.E.S.T. Medical Systems, Vienna, Austria) for further analysis. Patients in the NIRS and the control group had a cerebral NIRS oximeter probe placed on the left frontal head and a peripheral muscle NIRS oximeter probe placed on the right forearm within 6 h after birth. The NIRS sensors were repositioned after every 6 h. Interoptode distance was 4.0 cm for the cerebral optodes and either 3.0 cm in neonates >1,500 g or 2.0 cm in neonates <1,500 g for the peripheral muscle optodes.

Cerebral and peripheral tissue oxygenation (TOI) were measured continuously with a sampling rate of 2/s. The mean of cerebral TOI (cTOI) and peripheral muscle TOI (pTOI) were calculated every hour. After every hour changes of cTOI/pTOI ratio were calculated as a trend monitoring. An increase of cTOI/pTOI ratio of more than 5% within a 6-h interval was assumed to be out of range (18, 20–22) and a first sign of centralization (beginning shock).

In the control group, NIRS parameters were not visible and the infants were treated according to routine (“treatment as usual”). In the NIRS group, NIRS parameters were visible and neonates were treated according to predefined intervention guidelines.

According to these guidelines, an echocardiography was indicated, if cTOI/pTOI ratio increased > 5% within less than 6 h. Echocardiography was performed by neonatologists, who had been trained in neonatal echocardiography at the Division of Cardiology, Department of Pediatrcs, Medical University of Graz. Echocardiography was performed with a Vivid 7 Pro ultrasonic device (10 MHz sector transducer General Electric, Fairfield, CT, USA) including assessment of any form of severe structural heart disease, volume status, contractility, ejection fraction, superior vena cava (SVC) flow and ductal patency. If blood pressure was at the low side of the normal range (MABP higher but close to gestational age) and/or in echocardiography the attending neonatologist considered volume status/cardiac output to be low and/or SVC flow to be low (routinely ≤ 40 mL/kg per minute), the neonatologist had to consider the following interventions: (i) administration of fluid bolus of 10 ml/kg (normal saline) (23, 24), (ii) if ventilated: decrease of mean airway pressure (23, 25, 26), or (iii) in case of patent ductus arteriosus (PDA): medical treatment for DA closure (23).

## Duration

Near-infrared spectroscopy monitoring and predefined clinical interventions started within 6 h after birth and lasted for 24 h (i.e., 24–30 h after birth). Arterial blood pressure was measured for 48 h after study inclusion (i.e., 24 h during and 24 h after NIRS measurement and predefined interventions). Clinical follow-up was conducted until term age or until discharge, whatever occurred first.

## Outcome Measures

Demographic, perinatal data, respiratory support, main diagnoses, and infections were documented in each neonate. For the diagnosis of any infection, blood samples were taken from the neonate on the first and second day of life and CRP and leukocyte counts were determined. A blood culture was taken on the first day of life (either infants’ blood or cord blood). Infection was defined, when the neonate had clinical signs and a positive blood culture and/or a CRP value above the cutoff value of 10 mg/l.

For analyzes, mean values of arterial oxygen saturation (SpO_2_), HR, MABP, cTOI, and pTOI for each hour were calculated in each neonate. Primary outcome was burden of hypotension in %mmHg of MABP < gestational age and/or use of catecholamines during 48 h after initiation of NIRS measurements.

Secondary outcome measures were incidence of cerebral injury (IVH any grade or PVL any grade) and mortality until term age or until discharge. Cerebral ultrasound was performed at the beginning and end of NIRS measurements, at days 4, 7, and 14 and before discharge.

## Statistics

Data are presented as mean and standard deviation (SD) or median and interquartile range (IQR) for continuous data and absolute and relative frequency for categorical data, respectively. Differences between NIRS group and control group were analyzed using *t*-test or Mann–Whitney *U*-test for continuous data and Chi-square test or Fishers’s exact test for continuous data. A *p*-value of < 0.05 was considered statistical significant. Statistical analyses were performed using SPSS 24.0 (SPSS, Chicago, IL, USA).

## Results

**Figure 1:**
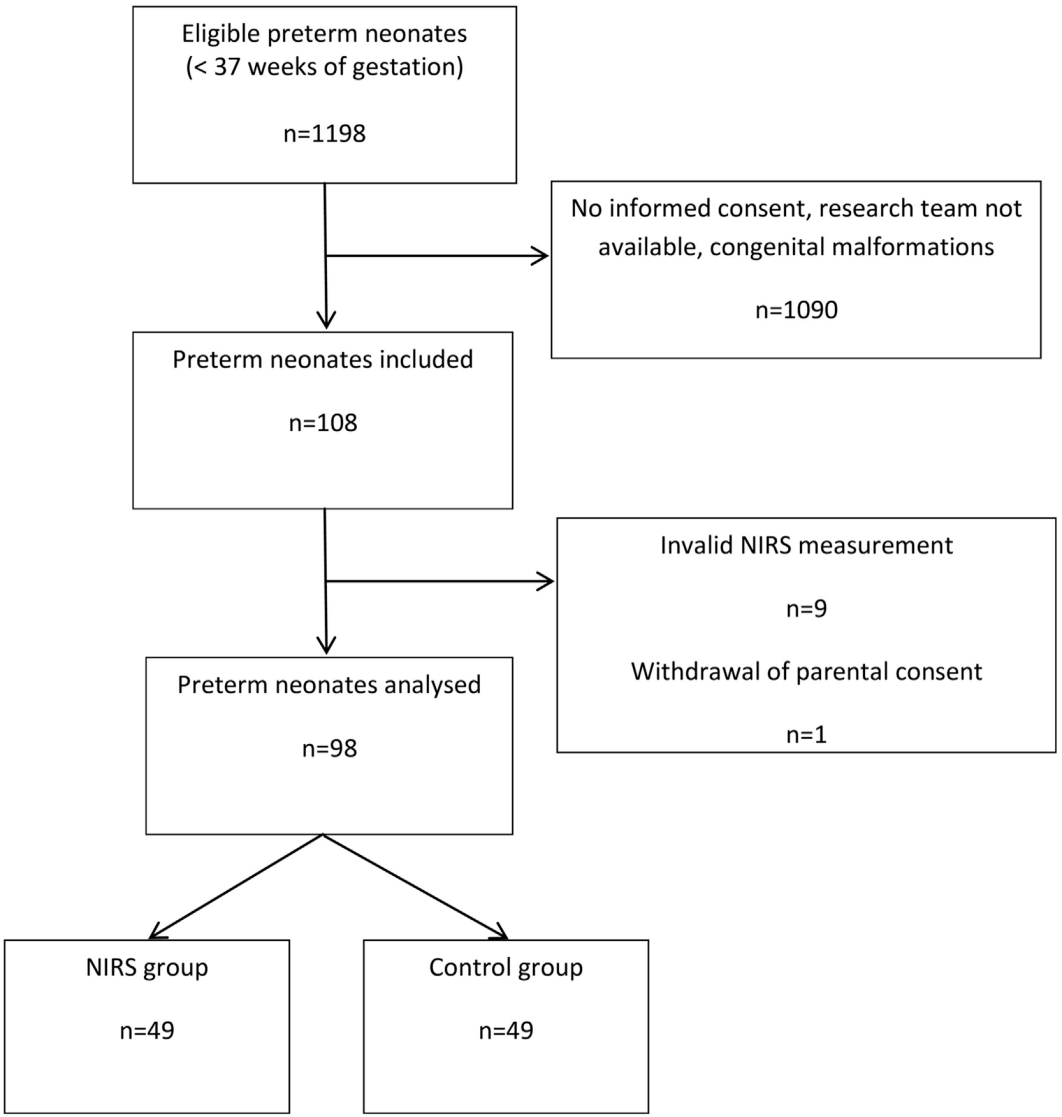
Flow diagram showing the number of included neonates and rationales for exclusion.

During the study period from October 2013 until December 2016 there were 1,198 deliveries of eligible infants < 37 + 0 weeks of gestation. 1,090 neonates (91.0%) were not enrolled mainly due to the research team not being available or lack of informed consent. In 108 neonates (9.0%), NIRS measurements were started. We excluded data of (i) eight neonates because measurements started beyond 6 h after birth, (ii) one neonate because no NIRS data were recorded, and (iii) one neonate because parental consent was withdrawn during measurements. Thus, 98 preterm infants (*n* = 49 in each group) were enrolled and analyzed (Figure 1). No severe adverse reactions were observed.

In the NIRS group, 20 mothers had premature rupture of membranes with/without infection and 29 mothers had primarily signs of infection/inflammation. In the control group, 22 mothers had premature rupture of membranes with/without infection and 27 mothers primarily signs of infection/inflammation. There were no significant differences in demographic characteristics between the groups (Table 1). Lowest birth weight in the NIRS group was 686 and 880 g in the control group. In the NIRS group, seven neonates were small for date and in the control group eight neonates. In the NIRS group, 25 neonates were admitted to the neonatal intensive care unit due to idiopathic respiratory distress (IRDS), 14 due to transitory respiratory distress of the newborn (TRDN), and 10 due to prematurity. In the control group, 23 neonates were admitted due to IRDS, 16 due to TRDN, and 10 due to prematurity. In the NIRS group, nine neonates were diagnosed with infection (according to criteria) compared to nine neonates in the control group. In each group, two neonates had culture proven infections. None of the neonates needed cardiocirculatory resuscitation with chest compressions. There were no significant differences in monitoring parameters including MABP between the groups (Table 2).

**Table 1:** Demographic and monitoring data of 49 preterm neonates with NIRS monitoring visible (NIRS group) and of 49 neonates with NIRS monitoring not being visible (control group).

	NIRS group (*n* = 49)	Control group (*n* = 49)	*p*-Value
Gestational age (weeks)	33.1 (32.0–34.0)	33.4 (32.3–34.3)	0.354
Birth weight (g)	1,826 ± 513	1,845 ± 504	0.856
Cesarean section, *n*	38	36	0.638
Male, *n*	26	23	0.544
Apgar 5 min	9 (8–9)	9 (8–10)	0.696
Umbilical artery pH	7.30 ± 0.04	7.29 ± 0.06	0.177
Respiratory support			0.699
No respiratory support, *n*	26	29	
Non-invasive respiratory support, *n*	21	17	
Intubated, *n*	2	3	
Surfactant, *n*	9	7	0.585
Infection, *n*	9	9	1.000

*Continuous data are presented as mean ± SD or median (25th percentile to 75th percentile) based on distribution*.

**Table 2:** Monitoring parameters and mean arterial blood pressure data of 49 preterm neonates with NIRS monitoring visible (NIRS group) and of 49 neonates with NIRS monitoring not being visible (control group).

	NIRS group (*n* = 49)	Control group (*n* = 49)	*p*-Value
Mean arterial oxygen saturation first day (%)	96.5 (94.0–98.0)	96.8 (95.5–97.6)	0.720
Mean heart rate first day (bpm)	144 (138–149)	141 (137–145)	0.192
Mean arterial blood pressure first day (mmHg)	44 ± 4	43 ± 5	0.918
Mean arterial oxygen saturation second day (%)	96.9 (95.3–98.0)	96.5 (94.4–97.5)	0.263
Mean heart rate second day (bpm)	144 (141–149)	142 (136–149)	0.245
Mean arterial blood pressure second day (mmHg)	46 ± 4	46 ± 5	0.773

*Continuous data are presented as mean ± SD or median (25th percentile to 75th percentile) based on distribution*.

Near-infrared spectroscopy measurements started earlier in the NIRS group (0.5 h) than in the control group. However, there were no significant differences in median cTOI and pTOI values between NIRS group and control group during the 24-h measurement period.

In the NIRS group, fewer alarms were recorded compared to the control group (Table 3). Seventeen neonates in the NIRS group (34.7%) were examined by echocardiography due to NIRS measurements. In two neonates, echocardiography revealed a PDA (diameter > 1.5mm/kg), which was treated with ibuprofen in both. In four neonates, a bolus of 10 ml normal saline/kg was administered due to echocardiographic low volume status.

**Table 3:** NIRS measurements and interventions of 49 preterm neonates with NIRS monitoring visible (NIRS group) and of 49 neonates with NIRS monitoring not being visible (control group).

	NIRS group (*n* = 49)	Control group (*n* = 49)	*p*-Value
Age at initiation, hours after birth	2.0 (1.5–3.5)	2.5 (2.0–4.0)	0.039*
Mean cerebral tissue oxygenation index (%)	70.5 (64.8–74.4)	71.2 (66.6–74.2)	0.548
Mean peripheral tissue oxygenation index (%)	73.7 (69.7–77.8)	72.6 (69.8–76.5)	0.354
Alarms/patient, *n*	3 (2–4)	4 (4–5)	0.008*
Echocardiographies, *n*	17	–	
Interventions, *n*	6	–	

*Continuous data are presented as median (25th percentile to 75th percentile) based on distribution*.

*p < 0.05.

In all, 32.6% of neonates in the NIRS group and 44.9% in the control group had hypotensive episodes, without significant differences either in number of neonates or in burden of hypotension (Table 4). No severe hypotension with need of catecholamine therapy was observed in both groups.

**Table 4:** Burden of arterial hypotension (%hours) of 49 preterm neonates with NIRS monitoring visible (NIRS group) and of 49 neonates with NIRS monitoring not being visible (control group).

	NIRS group	Control group	*p*-Value
48 h (during and after NIRS measurements)			

All neonates, *n* (%)	49 (100)	49 (100)	
Burden in all patients (% h)	0.0 (0.0–2.1)	0.4 (0.0–3.3)	0.313

Neonates with hypotensive episodes, *n* (%)	16 (32.6)	22 (44.9)	0.214
Burden in neonates with hypotensive episodes (% h)	3.0 (2.2–6.1)	4.2 (1.7–6.3)	0.804

Regarding cerebral injury, only four neonates had an IVH grade 1, one in the NIRS group and three in the control group. None of the included neonates died.

## Discussion

In the present study, simultaneous peripheral and cerebral NIRS monitoring followed by predefined interventions led to a non-significant reduction in burden of arterial hypotension in preterm neonates. Nonetheless, in six neonates in the NIRS group echocardiography due to a NIRS monitoring alarm was followed by a medical intervention.

The relevance of arterial hypotension and the indication for treatment is still under discussion. Several observational studies have demonstrated no benefit of antihypotensive therapy (27–29). Contradictory to these findings, an analysis of the EPIPAGE cohort just recently revealed that in extremely premature infants an antihypotensive treatment was associated with improved short-term outcomes (30). Thus, preterm neonates with arterial hypotension—defined as MABP being below gestational age in weeks—receive treatment at many neonatal intensive care units at the moment.

Dempsey et al. (31) reported that hypotensive preterm infants with clinical evidence of good perfusion have as good an outcome as normotensive patients, while treated low blood pressure was associated with adverse outcome. Hence, it may be beneficial to treat arterial hypotension especially in association with poor perfusion.

The aim of the present study was to recognize poor perfusion (compromised microcirculation) at an early stage, which then may help identifying neonates in need of cardiocirculatory support before macrocirculation is compromised. However, in the present study mainly late and moderate preterm neonates were included. This was responsible for the fact that most of the neonates were stable from a cardiocirculatory standpoint, which is the main limitation of the present findings. Neonates in both groups and especially in the control group only had borderline hypotension and none of the neonates needed inotropes at all. Therefore, effect of simultaneous peripheral and cerebral NIRS measurements followed by predefined interventions on prevention on severe arterial hypotension cannot be ruled out by the present data.

In the present study, echocardiography was performed by a neonatologist, when indicated in the NIRS group. The evaluation of the cardiovascular status of term and preterm infants by a neonatologist is gaining significant interest to support bedside clinical decision making. However, neonatologist performed echocardiography needs to be trained according recommendations to ensure standardization of training and clinical practice guidelines (32).

pTOI was slightly higher compared to cTOI in the present study. At first, this seems contradictory to recent findings. Grossauer et al. (33) described a ratio of cTOI/pTOI of 1.14. However, in her study term neonates with a mean age of 16 days were measured having similar cTOI with 70.4% and a much lower pTOI with 62.1% compared to present values. The different findings may be explained by a physiological decrease of pTOI during the first days after birth. pTOI has been described to decrease from 67 to 61% from the first to the third day after birth (20–22).

Recently we described that in stable preterm and term neonates with inflammatory processes peripheral oxygenation is lower compared to neonates without inflammatory processes (9). The mean pTOI value of the present study was within the range of recently published pTOI of neonates with and without inflammatory processes. This may be explained by the fact that neonates with and without inflammation/infection/sepsis were distributed equally in both groups.

Four neonates had a mild IVH and none of the included neonates died. These low numbers may be explained by the fact, that mainly moderate and late preterm neonates were included. Rapid, harmful alterations in blood pressure with hypo- or hyperperfusion have not been observed (34, 35). In addition, Binder et al. (36) demonstrated that mild short-term hypotensive episodes in preterm infants did not affect cerebral oxygen saturation, suggesting that cerebral autoregulation is maintained in case of borderline hypotension and may protect infants from cerebral injury.

## Limitations

Our main limitation is that most neonates included were moderate to late preterm neonates in cardiocirculatory stable conditions. Therefore, none of the neonates in the NIRS group and even none in the control group had severe arterial hypotension or was treated with inotropes. As a further fact of that inclusion none of the neonates died or had severe cerebral injury.

Near-infrared spectroscopy measurements started significantly earlier in the NIRS group compared to the control group. However, the observed difference was only 30 min and may not be of clinical relevance.

In the control group, the number of recorded potential alarms was higher compared to the NIRS group. Explanation might be that neonates with alarms and low arterial blood pressure in the NIRS group received treatment, causing a reduction in cTOI/pTOI ratio increase over time.

## Conclusion

In the present study, in preterm neonates simultaneous peripheral and cerebral NIRS measurements followed by dedicated interventions led to a non-significant reduction in burden of arterial hypotension. The present study did not prove that this approach resulted in significant better outcome, but that due to the trend these simultaneous peripheral and cerebral NIRS measurements may have the potential to become a clinical tool to recognize disturbances of peripheral microcirculation at an early stage before macrocirculatory impairment becomes evident. The described method should therefore be evaluated in more sick neonates with higher risk of arterial hypotension (e.g., neonates with proven sepsis or neonates in need of mechanical ventilation) and more preterm neonates.

## Ethics Statement

This study was carried out in accordance with the recommendations of Regional Committees on Biomedical Research Ethics with written informed consent from all subjects. All subjects gave written informed consent in accordance with the Declaration of Helsinki. The protocol was approved by the Ethikkommission, Medizinische Universität Graz, Austria.

## Author Contributions

Conception and design: GP, AA, and BU. Collection and assembly of data: GP, NH, NB-S, BS, LM, JS, and JP. Analysis and interpretation of the data: GP, NH, NB-S, BS, LM, JS, AA, JP, and BU. Drafting of the article: GP and AA. Critical revision of the article for important intellectual content: GP, NH, NB-S, BS, LM, JS, AA, JP, and BU. Final approval of the article: GP, NH, NB-S, BS, LM, JS, AA, JP, and BU.

## Conflict of Interest Statement

The authors declare that the research was conducted in the absence of any commercial or financial relationships that could be construed as a potential conflict of interest.

## Abbreviations

TOI, tissue oxygenation index; cTOI, cerebral tissue oxygenation index; pTOI, peripheral tissue oxygenation index; CRP, C-reactive protein; IVH, intraventricular hemorrhage; SVC, superior vena cava; SD, standard deviation; IQR, interquartile range; HR, heart rate; MABP, mean arterial blood pressure; NIRS, near-infrared spectroscopy; PDA, patent ductus arteriosus; SpO_2_, arterial oxygen saturation; TRDN, transitory respiratory distress of the newborn.
